# Electromagnetic Energy Redistribution in Coupled Chiral Particle Chain-Film System

**DOI:** 10.1186/s11671-018-2600-8

**Published:** 2018-07-05

**Authors:** Yuxia Tang, Yingzhou Huang, Linhong Qv, Yurui Fang

**Affiliations:** 1 Soft Matter and Interdisciplinary Research Center, College of Physics, Chongqing University, Chongqing, 400044 China; 2 Key Laboratory of Materials Modification by Laser, Electron, and Ion Beams (Ministry of Education), School of Physics, Dalian University of Technology, Dalian, 116024 China; 3 School of Computer Science and Information Engineering, Chongqing Technology and Business University, Chongqing, 400067 China

**Keywords:** Chiral plasmonic nanostructures, Electromagnetic energy focusing, Chiral near-field enhancement, Chiral focusing

## Abstract

Metal nanoparticle-film system has been proved that it has the ability of focusing light in the gap between particle and film, which is useful for surface-enhanced Raman scattering and plasmon catalysis. The rapid developed plasmonic chirality can also be realized in such system. Here, we investigated an electromagnetic energy focusing effect and chiral near-field enhancement in a coupled chiral particle chain on gold film. It shows large electric field enhancement in the gap between particle and film, as well as chiral near field. The enhancement properties at resonant peaks for the system excited by left circularly polarized light and right circularly polarized light are obviously different. This difference resulted from the interaction of circularly polarized light and the chiral particle-film system is analyzed with plasmon hybridization. The enhanced optical activity can provide promising applications for the enhancement of chiral molecule sensor for this chiral particle chain-film system.

**Electronic supplementary material:**

The online version of this article (10.1186/s11671-018-2600-8) contains supplementary material, which is available to authorized users.

## Background

Localized surface plasmon resonance (LSPR) resulting from coupled coherent collective oscillations of free electrons in metallic nanostructure and incident light associates strong electromagnetic near field around nanostructure. One of the main issues for LSPR is investigating the plasmonic nanostructure with nanometer-scaled gaps, in which one or more “hot spots” with high electromagnetic field enhancement arose. The hot spots make kinds of metal nanostructure be promising applied in various areas such as surface-enhanced Raman spectroscopy (SERS) [1–6], solar light harvesting and photo-catalysis [7–9], electron energy loss spectroscopy [10, 11], chemical and biological sensing [12, 13], surface-enhanced photoemission [14–18], nonlinear optics [19, 20], and photodetection [21, 22]. The field enhancement in plasmonic nanostructures with nanometer-scaled gaps, such as dimers [23–29], trimers [30–32], and other oligomers [33], has been studied and offered some flexible ways to tune optical properties of nanostructure by changing the excitation wavelengths, the size of oligomers, arrangement of nanoparticles (NPs), and inter-particle gap distance. It is worth noticing that the electromagnetic energy will redistribute because of the interaction of NPs and metal film in a complex NP-metal film system, and the system exhibits stronger field enhancement than general oligomers. Such coupled particle-film structures can potentially be applied in molecular spectroscopy [34–40].

In recent years, a lot of attention has been drawn to plasmonic optical activity, which is the different response of chiral plasmonic structures for left circularly polarized light (LCP) and right circularly polarized (RCP), even if the material itself is not chiral. One effect is named as circular dichroism (CD,$\uptheta =\left({I}_R^{\frac{1}{2}}-{I}_L^{\frac{1}{2}}\right)/\left({I}_R^{\frac{1}{2}}+{I}_L^{\frac{1}{2}}\right)\approx \Delta \mathrm{A}\left(\frac{\ln 10}{4}\right)$) which describes the extinction difference of LCP and RCP. Many biomolecules including amino acids, nucleic acids, and proteins exhibit CD effect, and CD analysis is essentially important on drug development, biomedicine, and life sciences. The CD response originating from interaction between chiral molecules and electromagnetic radiation is extremely weak, so the major investigation task is to enhance the resulting optical signal. Various plasmonic nanostructures have been explored, such as helical arrangement metal NPs [41, 42] and chiral metamaterials [43]. In these structures, the chiral nanostructures that are composed with achiral metal NPs exhibit huge optical activity originating from plasmonic interaction between achiral NPs [44–48]. And the hot spots in the near field of NPs are “super-chiral,” so called super-chiral fields [49–51], which can induce exceptional strong chiral near-field enhancement for exploiting chiral molecules and designing chiral optical device [52, 53]. However, most of the studies about super-chiral fields have focused on the enhancement between particle-particle in oligomer or oligomer-film system, the chiral near-field enhancement between particle-film is seldom considered. As above introduced, in fact strong near-field enhancement also occurs in the gap region between particles-film in the oligomers on a metal film system [34–40]. In consequence, for the complex particle-film system, there is strong super-chiral field not only between particles, but also between the particle-film gap, which can facilitate the measurement of chiral molecule samples with substrates. Strong CD responses benefit their potential applications in many fields such as detection of trace amount molecules [49], chiral discrimination [54], and polarization-sensitive optical devices [55].

In this work, we investigated the optical properties of a complex system consisting of chiral nanoparticle chain on gold film under LCP and RCP light excitation. The electromagnetic energy in the gap regions between particles and between particle-film exhibits different distributions, and the enhanced field results in super-chiral near-field and strong circular dichroism (CD) response. The huge chiral optical responses in the system may have promising applications in the detection of trace amount of chiral molecules.

## Methods

**Fig. 1:**
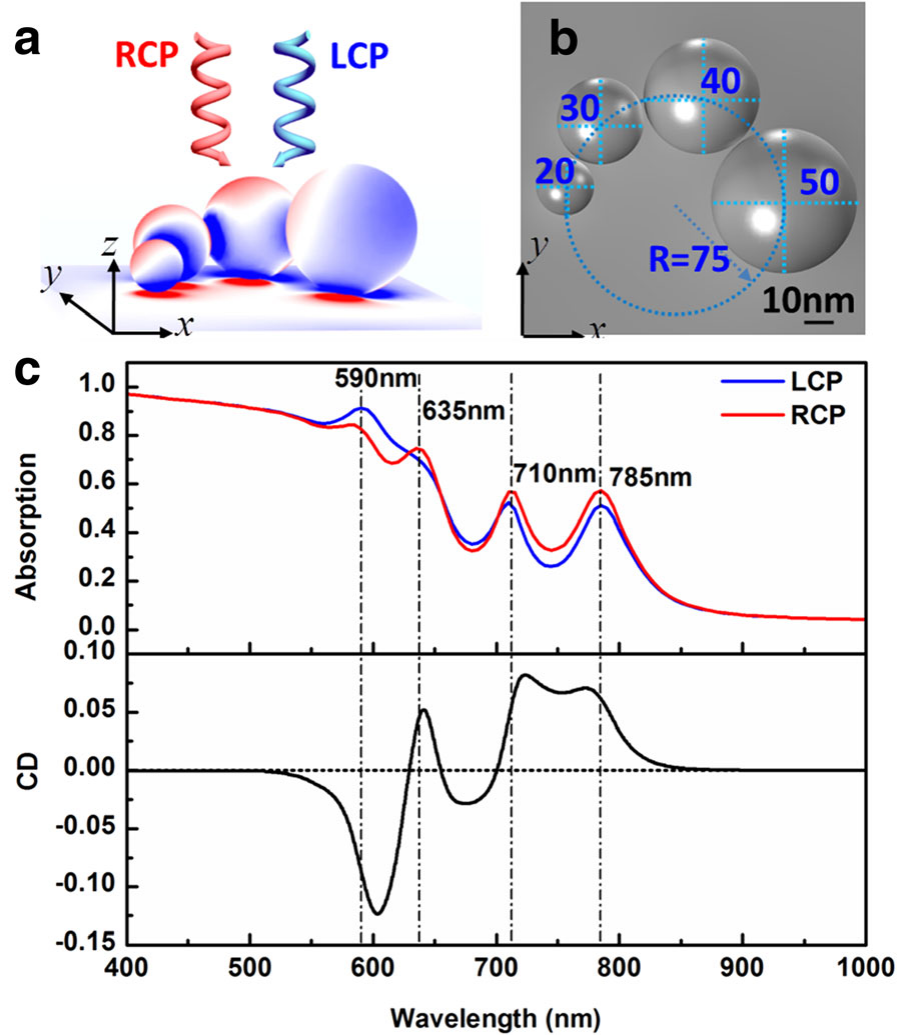
Excitation model of a chiral particle chain-film system and the optical properties. **a** Oblique view: The chiral particle chain is consisted of closely spaced Ag nanospheres with different radius of 20 nm (smallest), 30, 40, and 50 nm (largest) and is placed on a 100-nm-thick gold film with 1 nm gap. The circularly polarized light with left-hand circular polarization (LCP) or right-hand circular polarization (RCP) illuminates from the chiral particle chain side normal to the Au film. The surrounding medium of the Ag nanospheres is air. **b** Top view: The center of every particle of the chiral particle chain is on a same circle with radius *R* in the *x*-*y* plane (as shown with blue dotted line). The radius of every particle is shown with Arabic numbers in the figure and the unit is nanometer. The smallest spaces of each two adjacent particles are 2 nm. **c** Optical properties of chiral particle chain-film system. The blue and red solid lines represent the absorption spectra for RCP and LCP light, respectively (labeled as LCP and RCP). Lower panel: the corresponding circular dichroism (CD) spectrum is shown with black solid line. The vertical dashed lines from left to right correspond to the peaks at 590, 635, 710, and 785 nm, respectively

The optical properties of a tetramer complex system consisting of chiral nanoparticles chain on gold film (chiral particle chain-film system) were numerically investigated by utilizing COMSOL Multiphysics based on the finite element method (FEM). The chiral particle chain consists of four Ag nanospheres with different diameters arranged clockwise from small to big when looking along the direction of the incident light. The particle chain, whose radius are 20, 30, 40, and 50 nm (left-handed (LH) structure), is showed in Fig. 1a, b. The sizes are chosen because the resonance peaks are in the usual experimental range. The four nanospheres are arranged on the same circle in an *x*-*y* plane (as shown by blue dotted circle in Fig. 1b, where radius *R* is 75 nm). The chiral particle chain is put on a 100-nm-thick Au film with 1-nm gap between particle-film. The gaps between each two adjacent particles in the particle chain are 2 nm. The gap sizes are chosen as 1 nm in the main text analysis because it is a very typical thickness for monolayer of molecules adsorbing on particles. The purpose of the work is to give reference theoretical results to experiments that the chiral molecules adsorbing on particles for sensing. The results of other gap sizes are put in Additional file 1 for reference. The chiral particle chain-film system was excited with LCP and RCP, respectively, coming from the particle chain side normal to the Au film. Three-dimensional (3D) full-wave simulations were performed with periodic boundary conditions in the *x* and *y* directions. The relative permittivity of silver and gold was extracted from the experimental data reported by Johnson and Christy [56]. The surrounding medium of the particle chain was set to 1.0. Non-uniform meshes were used to format the object. The largest mesh was set to less than *λ*/6. The particle chain was placed in the *x*-*y* plane. The incident light was set to 1 V/m and propagated along the *z* axis. The reflective spectra (*R*) were obtained by the ratio of the reflected power flow and incident power flow. Because the sample is not transparent, we got absorption by 1 − *R*.

## Results and Discussions

### The Absorption Spectra and CD Spectra Analysis

The absorption spectra of the systems for LCP and RCP light are plotted with blue and red solid curves in Fig. 1c. It can be seen that there are four main plasmon resonant peaks, which are at around 590, 635, 710, and 785 nm. Comparing the two absorption spectra, there are obviously differences in the peak positions and the intensity of resonant peak in the range from 530 to 860 nm, which is particularly obvious in the two short resonant wavelengths. Figure 1c lower panel shows the CD spectra (CD ≈ ΔA = (1 − *R*_*R*_) − (1 − *R*_*L*_) = *R*_*L*_ − *R*_*R*_ in our system, *R*_*L*_ and *R*_*R*_ are reflectivity for the structure excited by LCP and RCP light respectively) of this system. We can see that there are stronger CD responses near the resonant peaks. Owing to the chiral arrangement of the particles, the responses of the four particles are different. The interactions between different responding particles will lead to a total response difference for LCP and RCP, which is chiral. The response can be explained by using matching or mismatching of the electric vector and the structure modes at specific moment. Very similar to a Born-Kuhn model, the rotating electric field vector will match different modes in different moment in a period of LCP and RCP and the electric vectors of LCP and RCP are rotating in counter directions [57, 58], which has also been proposed in several previous works [42]. However, in the condition of the particle chain system, the imaging charges on the film will interact with the particle chain and form an equivalent double chain. In consequence, there are strong CD responses because of the rotating electric vectors of incident LCP or RCP light that is along or counter to the dipole direction formed by the LH particle chain on film at specific excitation wavelengths.

The comparison of absorption spectra and circular dichroism spectra for different gaps between particles are put in Additional file 1: Figure S1 to help showing the trend. We can see that when the gap becomes smaller, the CD becomes stronger, which is not surprising because of the stronger interactions.

### Circularly Polarized Light Focusing Analysis

**Fig. 2:**
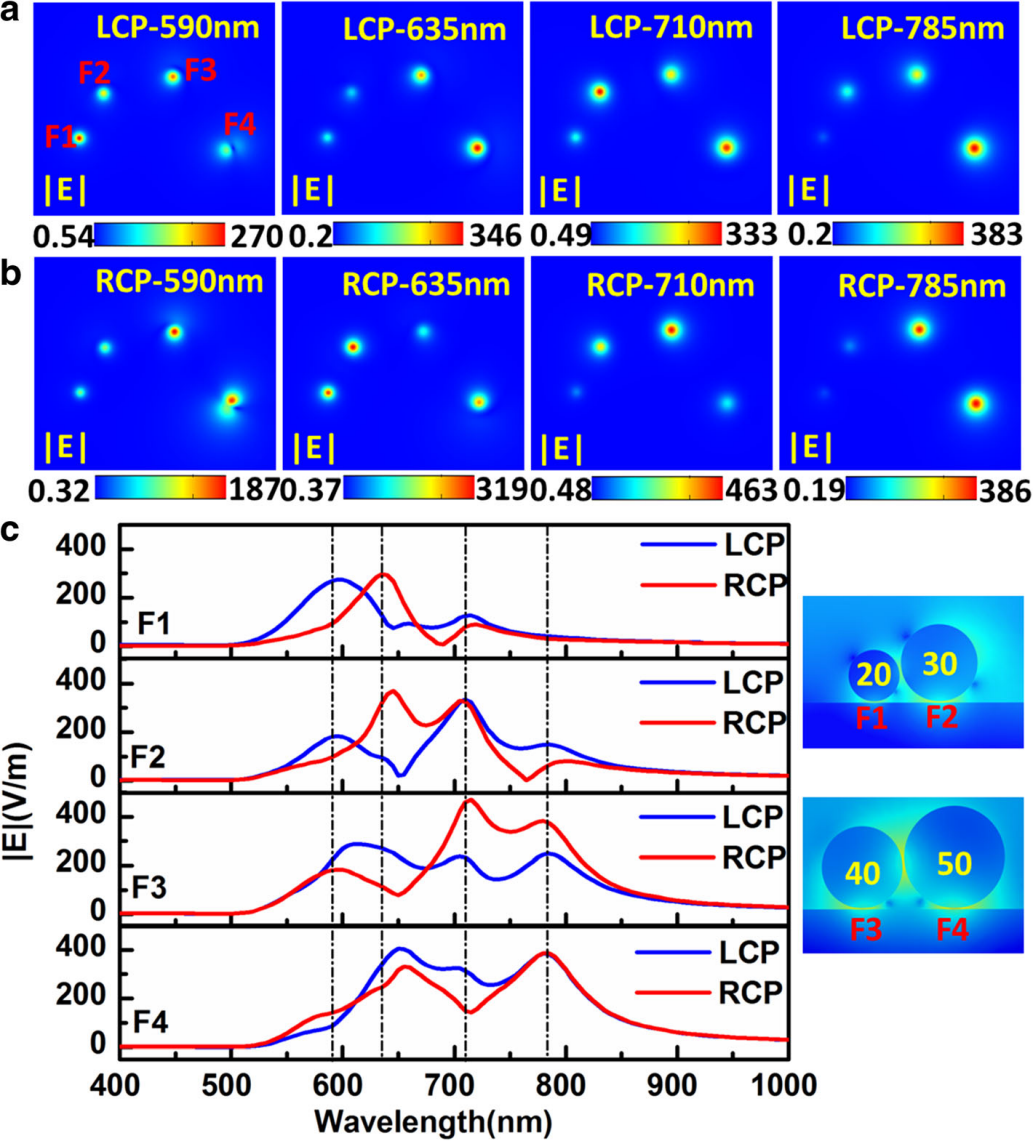
The electromagnetic energy focusing effect in the Ag chiral particle chain-film system. **a**, **b** The electric near-field distributions in the *x*-*y* plane in the middle of gap between chiral particle-film at different resonant peaks for the system excited by LCP (**a**) and RCP (**b**) light, respectively. Each picture corresponds to the marked resonant peak. **c** Electric field enhancements in the gaps F1, F2, F3, and F4, indicated in the right pictures (and in **a** as well) show huge differences at resonant peaks. The blue and red solid lines represent electric field enhancement for LCP and RCP light. The vertical dashed lines from left to right correspond to the peaks at 590, 635, 710, and 785 nm, respectively

Our previous studies have shown that “hot spots” with high electromagnetic field enhancement for the nanoparticle-metal film system occurs not only between nanoparticles but also between nanoparticle and metal film. And in some instances, the electromagnetic field between particle and film is even stronger [35, 36]. The electromagnetic energy would redistribute because of the different interactions between chiral particle chain-film and LCP/RCP exciting lights. The near-field energy focusing effect of the system is investigated for difference circularly polarized light, as shown in Fig. 2. Figure 2a, b displays the electric field distribution in the middle of the gap between the chiral particle chain and the Ag film at the resonant peaks for LCP and RCP light, respectively. For convenience, the gaps between different diameter particles and film are labeled as F1, F2, F3, and F4 (as shown in the right column of Fig. 2c. In Fig. 2a, b, every graph represents the electric field distribution at corresponding resonant peak, and each field enhancement position respectively corresponds to F1, F2, F3, and F4. At the same excited wavelength, the field enhancement position and the intensity exhibit obvious difference for LCP and RCP. At 590, 635, 710, and 785 nm resonant wavelengths, the strongest fields occur at F1, F4, F2, and F4, respectively. For LCP, the corresponding maximum field enhancements are 270, 346, 333, and 385, respectively. However, the strongest fields occur at F3, F2, F3, and F3–F4 at above wavelength for RCP; and corresponding maximum enhancements are 187, 319, 463, and 386, respectively. In addition, the field enhancements of other gaps also show diversity for different wavelength for LCP and RCP. Figure 2c shows the electric field intensity in gaps between different particles-film in the chiral particle chain-film system with the exciting wavelength varying from 400 to 1000 nm. The blue solid curve represents the electric field for LCP and the red solid curve for RCP. Roughly, the maximum enhancement occurs at smaller particle with shorter wavelength resonance peak and at larger particle gap with longer wavelength resonant peak, which is in accordance with expectation but not an absolutely tending. In addition, in the same gap with different resonant peaks, or in different gaps at the same resonant peak, the near-field enhancements also show significant differences in the positions of resonant peak and intensity of enhancement for different circular polarized light. In the gaps of F1 and F2, the maximum enhancement difference caused by different polarity of LCP light and RCP light occurs around 635 nm resonant wavelength, and the ratios of the enhancements under RCP and LCP are 3.5 and 5.5 for F1 and F2, respectively. For F3, the larger enhancement differences are founded around 635 and 710 nm, and the enhancement ratios for LCP to RCP respectively are 3 and 0.5. It is worth noting that the 0.5 times enhancement here shows stronger enhancement under RCP light than LCP light around 635 nm. For F4, there is largest enhancement difference around 635 nm, and the ratio of LCP to RCP is 1.4. These phenomena are good for exciting CD response and provide prospects of molecular sensor on substrate at different positions.

**Fig. 3:**
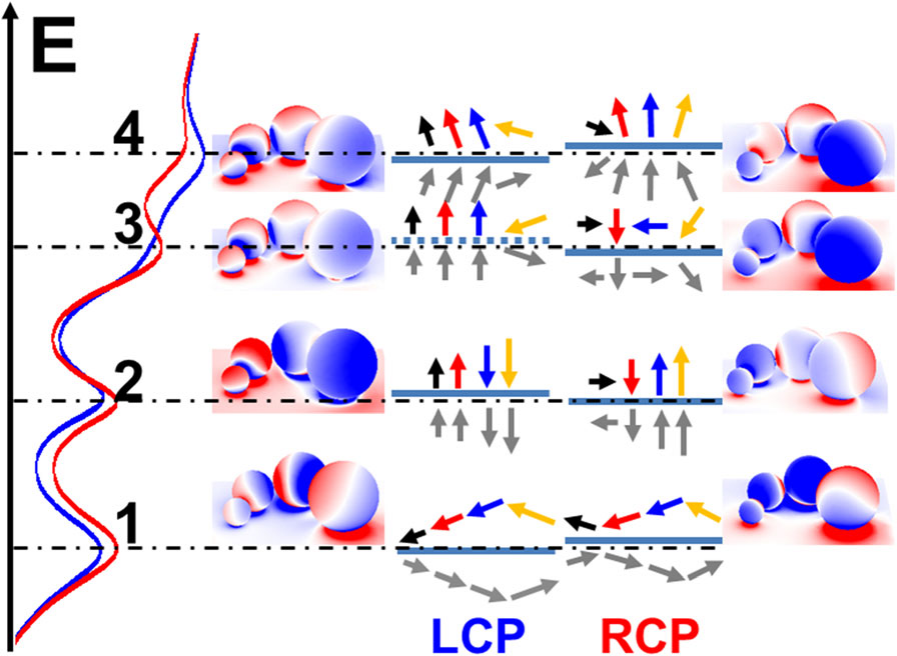
Scheme of the hybridization of the chiral particle-film system. The blue and red solid curves on the left are the reflectance spectra for the system excited by LCP and RCP, respectively. The surface charge distributions of the chiral particle chain and the gold film for LCP or RCP light are shown on left and right sides. The color arrows indicate the polarization states of the different radius particles, which are 20, 30, 40, and 50 nm for the black, red, blue, and yellow. The horizontal dashed lines from up to down represent the energy levels of the peaks at 590, 635, 710, and 785 nm, respectively

To get a deep understanding of the mechanism behind the electric field enhancement of the chiral particle chain-film system, we now investigate the mode of each resonant peak from a hybridization point of view. According the reflectance spectra of the system, there are four resonant peaks which are marked as 1, 2, 3, and 4 respectively, as shown in Fig. 3 (the blue for LCP and red curve for RCP). The surface charge distributions of the system under LCP or RCP excitation are shown on the left and right, respectively, with the hybrid levels in the middle. The color arrows indicate the polarization states of the different radius particles, which are corresponding to 20, 30, 40, and 50 nm plotted with black, red, blue, and yellow colors. The film is represented as the cyan line under arrows; and the cyan lines represent the hybrid levels as well. The induced opposite imaging dipoles on the film are shown with gray color.

For the first level around 785 nm, it is a typical hybrid large dipole mode with induced imaging opposite dipole on the film. For the RCP excitation, because of the weaker moment for the 50 nm particle (yellow dipole), the energy level blue shifts several nanometers (one need to look very closely to resolve it) related to the LCP excitation. According to the hybridization theory of particle and film [34], one knows that for such induced imaging charge coupling, the system is always a bonding mode, so that it always lowers the energy. If the coupling is weaker, the energy will be higher (blue shift). From the coupled large dipole and imaging dipole, we can easily see that for the LCP excitation, the strongest electric field should be under the 50 nm particle (yellow dipole); and for the RCP excitation, the strongest points F3 and F4 should be under 40 nm (blue) and 50 nm (yellow) particles (50 nm is even stronger). For the second level around 710 nm, from the surface charge distributions on the particles, we can see that for the LCP excitation, the smaller two particles are in the same orientation (which is an antibonding mode for the two particles) [59] and are vertical to the surface; the larger two particles are in the same orientation with opposite direction to the smaller two. But for RCP excitation, the black dipole polarization is in horizon, which will lower the level energy. From the dipole directions, one should consider that for LCP excitation the red and blue dipole are opposite, so they can strengthen each other so that F2 and F3 should be stronger. According to Ref. [36], in this case, the smaller particle will confine more energy, so F2 is the strongest gap. For RCP excitation, the horizon one (black arrow) cancels F2 partially because the black dipole and the red dipole are partially antibonding, so F3 is the strongest. The third level is around 635 nm. Compared to the second level, the blue dipole flips over, and the strong field yield by the black, red, and blue dipoles draw the yellow dipole in a titled angle. The flipped blue dipole also makes the level energy higher under LCP excitation because this mode is hybridized by the three dipoles. When excited with RCP, the black and blue dipoles are horizon making the energy lower. From the dipoles’ orientations, we can directly find that the gap F4 is the strongest for LCP because the blue arrow and yellow arrow strengthens each other and F2 is the strongest for RCP because the black and blue arrows strengthen the red one. For the fourth level around 590 nm, the almost same orientated four dipoles make the energy the highest. Under LCP excitation, the first three dipoles are almost in the same orientation, and the yellow one is titled in a large angle, which lowers the energy. Under RCP excitation, the titled dipole is the black one. Because the dipole moment is weaker, the total energy is higher than the LCP execution one. From the dipole configuration, we can see that for the LCP, the strongest gap should be F1 under the smallest particle because the smallest particle has the strongest focus ability under such configuration because the larger particles will enhance the field around smaller one more. For the RCP, the strongest gap is not F1 or F2 because the black dipole is too small and it is almost under the gap of the red dipole. So the opposite field enters the red dipole’s gap too much and cancels the field. For the blue and yellow ones, the strongest gap should be F3 because the blue particle is smaller. In total, the analysis in the hybridization fits the result in Fig. 2 very well.

**Fig. 4:**
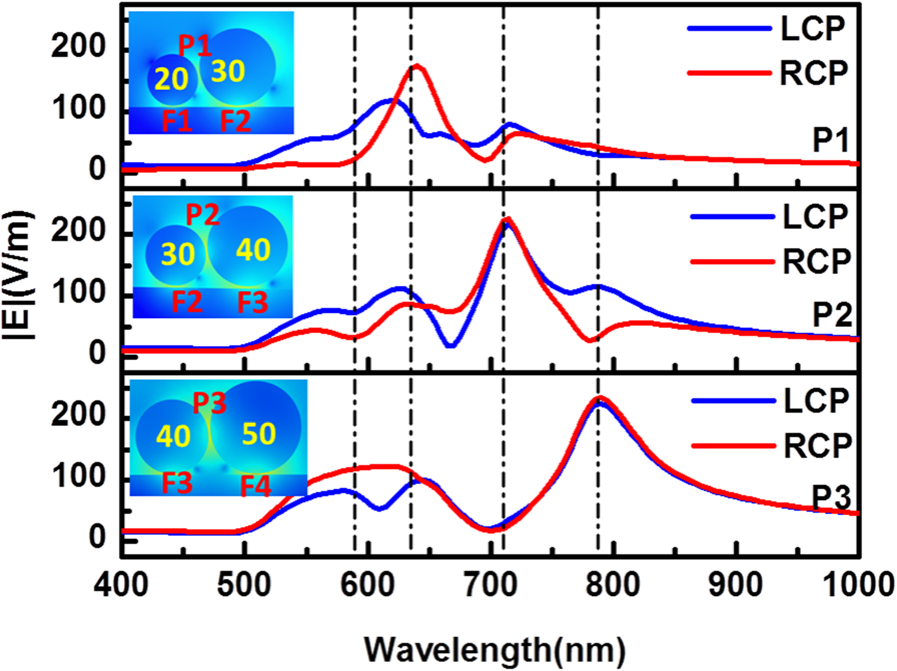
The electric field enhancement in the gaps P1, P2, and P3 (as indicated in the insets) between different silver particles in the chiral particle chain. The blue and red solid lines represent the electric field enhancement spectra for LCP and RCP light, respectively. The vertical dashed lines from left to right correspond to the peaks at 590, 635, 710, and 785 nm, respectively

In fact, the electric field enhancement between the particles in the chiral particle chain-film system is also very strong. The enhancement spectra in gaps P1, P2, and P3 between particles (insets of Fig. 4) are shown in Fig. 4. We note the maximum field peaks shift towards long wavelength when the diameter of adjacent particles increases, which are at about 620 nm for gap P1, 710 nm for gap P2, and 785 nm for gap P3, respectively. The maximum electric field enhancements are 120, 217, and 226. The trend is similar to RCP exciting. It is interesting that the enhancement peak positions are not exactly same for LCP and RCP excitation. Nonetheless, compared to the electric field enhancement spectra shown in Fig. 2c, the enhancement effect between particles-film is stronger than that between particles.

### Super-Chiral Field in Chiral Focusing

**Fig. 5:**
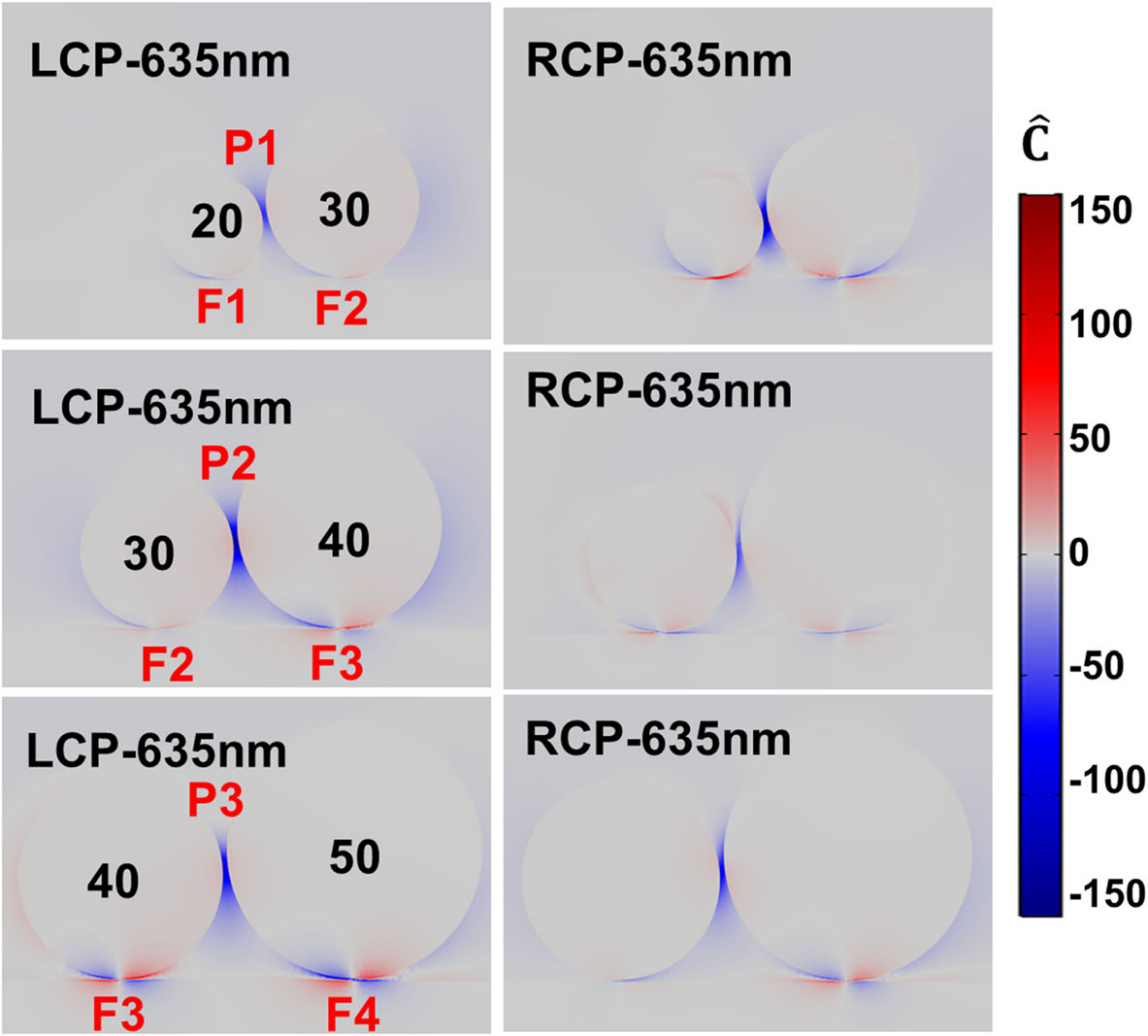
Chiral near-field enhancement distributions in the gaps between particles and between particles-film at 635 nm resonant peak for LCP and RCP excitation. The diameters of four silver nanospheres in chiral particle chain-film system are respectively marked as 20, 30, 40, and 50 nm. The gaps between different diameter particles and film are respectively labeled as F1, F2, F3, and F4, and the gaps between particles are labeled as P1, P2, and P3, respectively

**Fig. 6:**
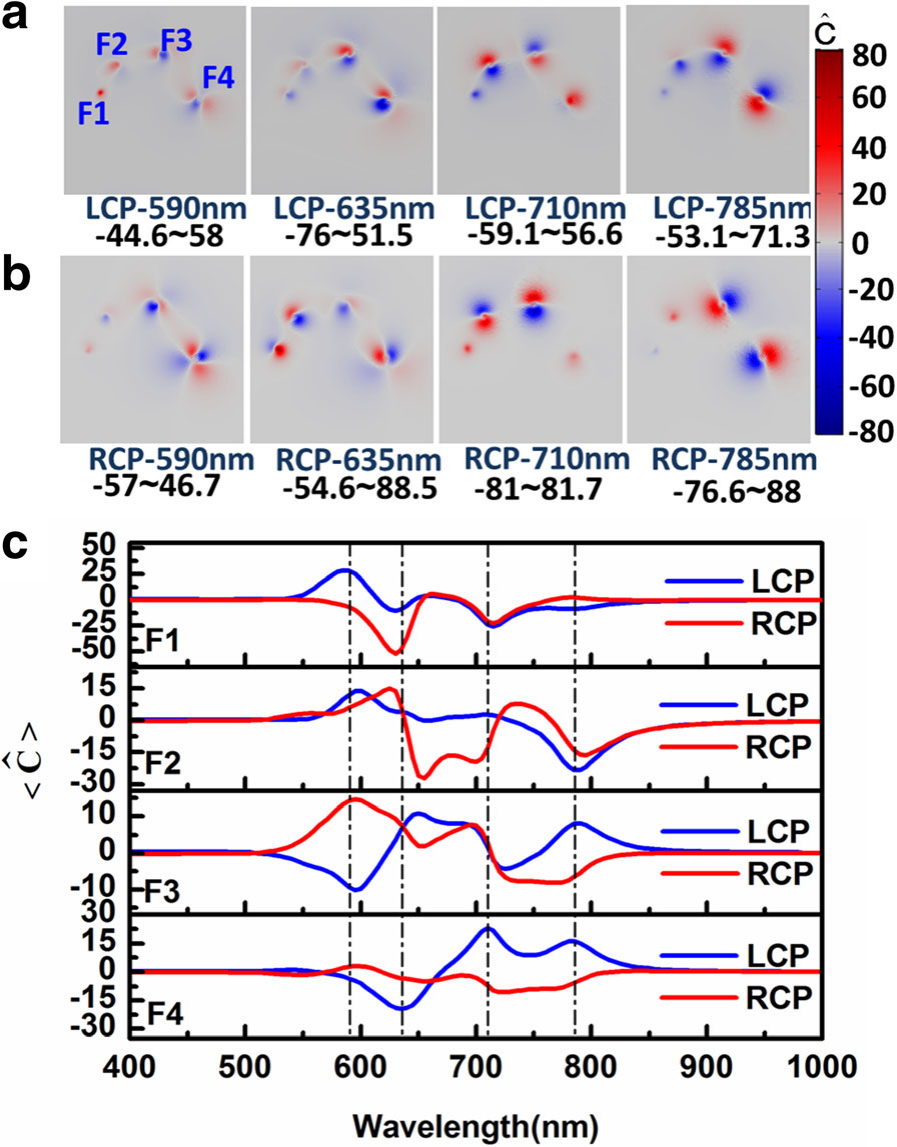
The optical chirality enhancement factors of the chiral silver particles chain on a gold film. **a** Chiral near-field enhancement distributions at the middle of the gap between particle-film in *x*-*y* plane at different resonant peaks for LCP light and **b** for RCP light. **c** Volume-averaged optical chirality enhancement factors in the gaps F1, F2, F3, and F4. The blue and red solid lines correspond to LCP and RCP light, respectively. The vertical dashed lines from left to right correspond to the peaks at 590, 635, 710, and 785 nm, respectively

It is very clear that once there is film under particles, there is focusing effect in the gap between the particles and film. And the focusing effect varies under LCP and RCP excitation. One of the big issues in plasmon-enhanced chirality is that the structure can yield super-chiral near fields, which is expected to be applied for sensing or detecting chiral molecules. To investigate the enhanced chiral near-field response, the super-chiral near-field enhancement around the systems is calculated. The optical chirality enhancement factor, which is defined as $\widehat{C}=C/{C}_{CP}$ [46], where *C* =  − *ε*_0_/2*ω* Im[***E***^∗^ ⋅ ***B***] is named optical chirality, as introduced by Tang and Cohen [50], which can be quantitatively characterized by the degree of chiral asymmetry. Here, *ε*_0_ is the permittivity of free space, *ω* is the angular frequency of the incident light, and ***E*** and ***B*** are the local electric and magnetic fields. ${C}_{CP}=\pm {\varepsilon}_0\omega {E}_0^2/(2c)$ is the optical chirality for the LCP light (+) and RCP light (−) with electric field amplitude *E*_0_. Because of the strong near-field coupling, the huge field enhancement in the gaps between particle-film (see Fig. 2) may generate obviously enhanced local chiral fields in the corresponding gaps. The local chiral field distributions at the resonant peak with 635 nm are shown in Fig. 5. We can find there is strong chirality enhancement in the gaps F4 and F3 for LCP. However, the enhancements occur in the gaps F2 and F1 for RCP. The above results are corresponding to Fig. 2. The local chiral field distributions for other resonant peaks are presented in Additional file 1: Figure S2 (a)–(c). In Fig. 6a, b, we show the optical chiral field enhancement distribution in *x*-*y* plane at the middle of the gap between particles-film at the four resonant peaks respectively when the system is excited by LCP and RCP light. The enhancement positions correspond to the regions of gaps F1, F2, F3, and F4 in clockwise, respectively. The strength of the enhancement in different gaps is different at same resonant mode for LCP and RCP light. Under the same CPL excitation, the super-chiral field in the gap also varies. We can see the chiral enhancement factor can reach 90 times at beneficial positions and resonant wavelength. The chiral optical enhancement is also selective for LCP and RCP light at the different locations of the same gap. In addition, the chiral enhancement regions are limited to a small area in each gap and rapidly change. In the chiral enhancement applications, CD signal over the probe volume is determined by the integration of the local chiral field. So it is necessary to investigate averaged optical chirality. Here, we take a small cylinder with a radius of 4, 6, 8, and 10 nm, respectively, under the particle with a radius of 20, 30, 40, and 50 nm, and the heights are just cutting the film and the particles. Each cylinder intersects with the corresponding particle and the film. The volume of difference region between the cylinder and every particle-film is *V*. The radius is chosen when the electric field decays to 1/e of the maximum under the particles. The averaged optical chirality enhancement factor can be obtained by integrating *C*/|*C*_*CP*_| in the differential part of the cylinder with the particle and the film and taking the average of volume, which is

1 $$ \left\langle \widehat{C}\right\rangle =\frac{1}{V}\underset{V}{\int }C/\left|{C}_{CP}\right| dV $$

and the corresponding volume-averaged chiral enhancement spectra in gaps F1, F2, F3, and F4 are plotted in Fig. 6c. From the spectra, we can see that even if there is a cancel effect for opposite chiral field under the particles, the averaged chiral field enhancement is still very strong. For F1, the strongest peak even reaches 50 times. The chiral field enhancement on metal film is rarely reported. The averaged chiral field enhancement on metal film in our work is with the same order as in ref. 51. And in different gaps, the chiral field under LCP and RCP excitation can be totally opposite, like in gaps F1 and F3. With the properties, we can use it for sensing chiral molecules. For example, when two chiral molecules with opposite chirality are located under gap F1, the *L* one will be excited under LCP at 590 nm and the *R* one will be excited under RCP at 635 nm. If one only excites at 600 nm, LCP and RCP light will totally distinguish the *L* and *R* molecules in gap F3.

**Fig. 7:**
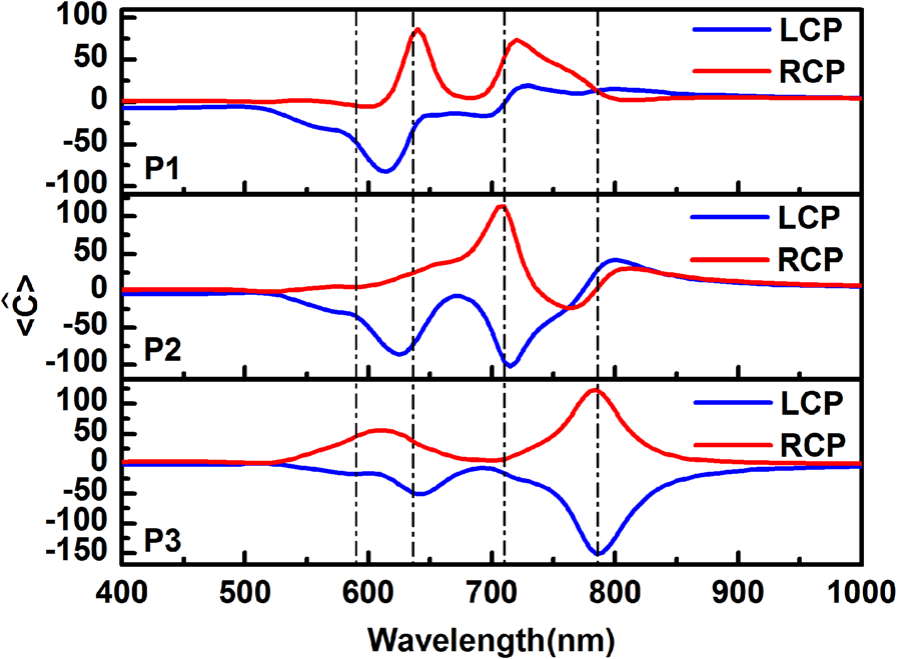
Volume-averaged optical chirality enhancement factors in the gaps P1, P2, and P3. The blue and red solid lines correspond to LCP and RCP light excitation, respectively. The vertical dashed lines from left to right correspond to the peaks at 590, 635, 710, and 785 nm, respectively

In addition to the strong chiral near-field enhancement in the gaps between particle-film, there are also large chiral responses in the gaps between particles (Fig. 5 and Additional file 1: Figure S2 (a)–(c) exhibit the chiral near-field enhancement distributions at resonant peaks in the gaps P1, P2, and P3). To view the chiral fields between particles, the average optical chirality enhancement spectra are also calculated by Eq. (1) in the regions of gap P1, P2, and P3, as shown in Fig. 7. The volume in the formula here is obtained with similar method as the particle-film gap. It is clearly observed the chiral fields in gaps P1, P2, and P3 are always negative for LCP in the wide wavelength range; for RCP light, the chirality of the field is opposite in the gaps. The significant difference for two circular polarized lights is important to the application of chiral molecular enhancement.

### Comparison with Linearly Arranged Particles, Larger Radius of Arrangement Circle, and Different Particle Number

**Fig. 8:**
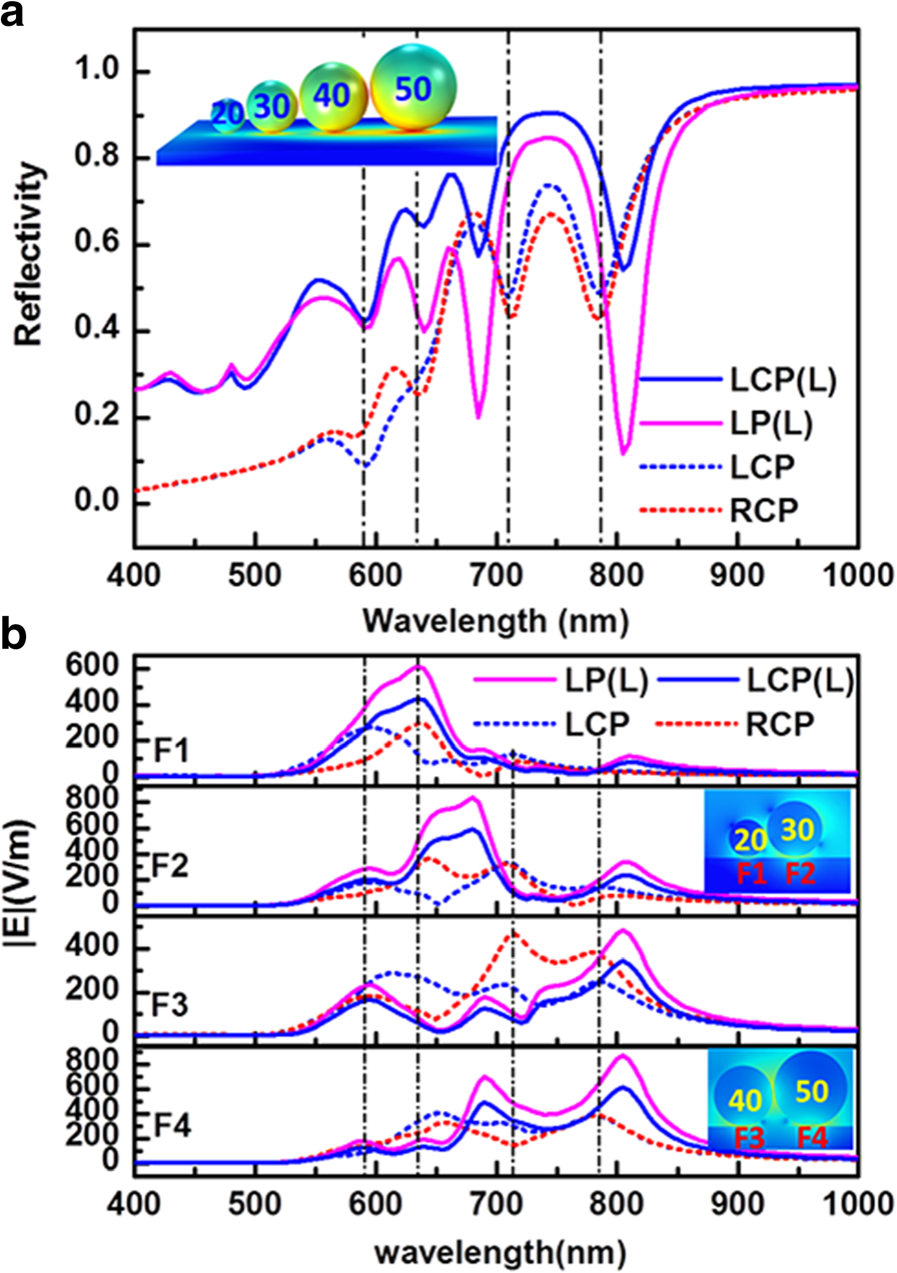
Comparison with linearly arranged particles by different polarized light. **a** Reflectance spectra of different silver particle-film system. The blue and pink solid lines represent the reflectance spectra of the linear silver particle chain-film system excited by left-hand circle light (marked as LCP(L)) and linear polarized light (marked as LP(L)), respectively. The structure of linear particle chain-film is shown in the inset. In the diagram, Arabic numerals 20, 30, 40, and 50 represent the radius of corresponding particles. **b** The electric field enhancement spectra in the gaps between particle-film in the chiral particle chain-film and the linear particle chain-film systems. The gaps between particle-film are respectively labeled as F1, F2, F3, and F4, which are indicated in the insets. The blue and pink solid lines represent the enhancement on the film in the linear chain structure excited by left-hand circle light (marked as LCP(L)) and linear polarized light (marked as LP(L)), respectively. The blue and red dotted lines are electric field enhancement for the chiral particle chain-film system probed by LCP and RCP light, respectively. The vertical dashed lines from left to right correspond to the peaks at 590, 635, 710, and 785 nm, respectively

**Fig. 9:**
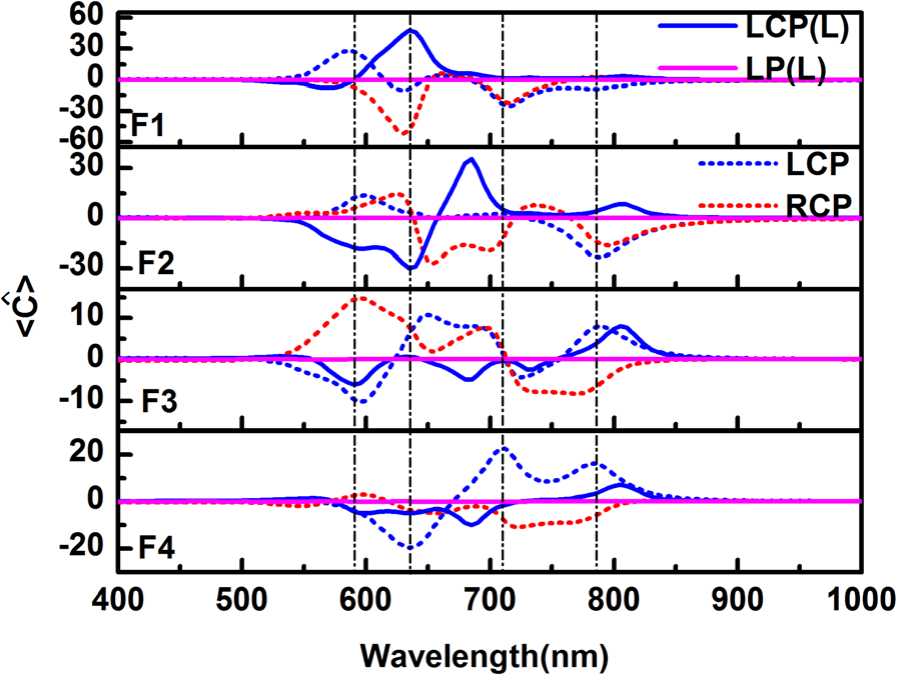
Volume-averaged optical chirality enhancement factors in the gaps F1, F2, F3, and F4 indicated in the insets of Fig. 8. The blue and pink solid lines represent the volume-averaged chiral near-field enhancement spectra of the linear structure excited by left-hand circle light (marked as LCP (L)) and linear polarized light (marked as LP (L)), respectively. The blue and red dotted lines represent the volume-averaged chiral near-field enhancement spectra of the chiral silver particle chain-film for LCP and RCP light, respectively. The vertical dashed lines from left to right correspond to the peaks at 590, 635, 710, and 785 nm, respectively

The electric field energy focusing effect of the linear particle chain on gold film system was also studied as a comparison. The linear particle chain is also consisted of four Ag particles with different diameters, which are 20, 30, 40, and 50 nm as shown in Fig. 8. In contrast to chiral structures, linear particle chain has a stronger reflex response (Fig. 8a), the focus effect in the gaps between particles-film is more pronounced, and especially the linear system is probed by linear polarized light (Fig. 8b). In Fig. 9, the volume-averaged chiral enhancement spectra in the gaps between particles-film are plotted. The solid curves represent the enhancement spectra of the linear particle chain-film system, and the blue and pink solid lines correspond to LCP light and linear polarized (LP) light, respectively. The dotted lines represent the enhancement of chiral particle chain-film system as above discussed, and the blue and red dotted lines correspond to LCP and RCP light excitation, respectively. One can notice that there is stronger chiral field enhancement in some gaps at some resonant peak, e.g., in gap F1 the chiral field enhancement may reach values of 48 near the peak 640 nm. However, compared to chiral structure, it is clear that for circular polarized light the optical chirality of the linear structure is weaker in general, and for linear polarized light the linear structure do not exhibit CD response, so it is more advantageous that chiral structure is used in chiral molecular sensor than linear structure.

To investigate the arrangement effect of the chirality of this structure on optical chirality, we changed the arranged circle radius (*R*) in *x*-*y* plane (as shown by blue dotted circle in Fig. 1b) and simulated the optical properties of the chiral NP chain-film system with different *R*. From the absorption spectra and CD spectra, we can see that the resonant peaks are almost the same with the increase of *R* (Additional file 1: Figure S3); but the CD becomes weaker when *R* increases. Because the chirality of structure becomes lower (symmetry becomes higher) with *R* increasing, the chiral responses for LCP and RCP are not so sensitive any more. Meantime, the volume-averaged chiral enhancement between NPs-film shows a trend of decrease as well (Additional file 1: Figure S4). However, *R* has less influence on the volume-averaged chiral enhancement between particles (Additional file 1: Figure S5).

In addition, the relation of chiral enhancement and particle number is also investigated (Additional file 1: Figure S6). Very similar with the above discussed system with four particles (which is labeled as 50-40-30-20), we took away the 20-nm particle to make the chiral chain having three particles (labeled as 50-40-30). The CD spectra of the two systems are obviously different. There are three CD response peaks in the 50-40-30 system. With the particle number reducing, the volume-averaged chiral enhancement in gap F2 is more affected than in gaps F3 and F4. However, the volume-averaged chiral enhancement between particles has small change.

The results presented above somehow give a way of enhancing chiral molecule optical activity signals other than direct enhancement by a dimer. However, to fabricate such system is a bit tricky. A rough way to make such system may be to directly drop a droplet particle sol with different size on the Au film substrate. Because there are plenty of particles, it is not very hard to find such curved shape with different size. But if someone want it more controllable and delicate, chemical synthesis is a possible way. The nanoparticle is not perfect round because of the crystalline structure. One can first put the particles with the uniform size in some functional molecule solution (like DNA with special functional group), in which the chemical molecules will only adsorb on specific facet. Perform similar steps on the particles with different size [11]. Mix the particles together and they will form a chain. Then, drop the solution on substrate and the tension of the solvent will curve the chain. Other possible way may be pulling method with mill curved slots substrate [60], magnetic self-assembly of particles with magnetic core particles [61], capillary effects [62], or optical force [63].

## Conclusions

In conclusion, we have demonstrated an electromagnetic energy focusing effect and chiral near-field enhancement of the chiral chain consisted of four different diameter nanoparticles on gold film. When the chiral chain is excited by LCP and RCP light, obvious difference electric field enhancement gaps are observed at resonant peak. The hybridization analysis recovers the mechanism. This difference in electric field enhancement results in strong chiral near-field enhancement near the gap between particles and between particle-film, which induces strong chiral response and provide prospect for chiral near-field enhancement applications in chiral molecule detection.

## Additional file

Additional file 1: **Figure S1**. The optical properties of the studied system with different gaps between nanoparticles; **Figure S2**. Chiral near-field enhancement distributions for different resonant peaks; **Figure S3**. The optical properties of chiral particle-film system with different R; **Figure S4**. The volume-averaged chiral enhancement spectra between NPs-film in chiral particle-film system with different R; **Figure S5**. The volume-averaged chiral enhancement spectra between particles in chiral particle-film systems with different R; **Figure S6**. Optical properties of chiral particle chain-film systems with different particle numbers. (DOCX 1098 kb)

## Abbreviations

CD: Circular dichroism
FEM: Finite element method
LCP: Left circularly polarized light
LH: Left-handed
LP: Linear polarized
LSPR: Localized surface plasmon resonance
NPs: Nanoparticles
RCP: Right circularly polarized light
SERS: Surface-enhanced Raman spectroscopy
