# Supplementary material for: Electromagnetic Energy Redistribution in Coupled Chiral Particle Chain-Film System
Source: Nanoscale Res Lett. 2018 Jul 5;13:194. doi: 10.1186/s11671-018-2600-8 (PMC6033841; doi:10.1186/s11671-018-2600-8)
Supplement: Supplementary file 1 — Figure S1. The optical properties of the studied system with different gaps between nanoparticles; Figure S2. Chiral near-field enhancement distributions for different resonant peaks; Figure S3. The optical properties of chiral particle-film system with different R; Figure S4. The volume-averaged chiral enhancement spectra between NPs-film in chiral particle-film system with different R; Figure S5. The volume-averaged chiral enhancement spectra between particles in chiral particle-film systems with different R; Figure S6. Optical properties of chiral particle chain-film systems with different particle numbers. (DOCX 1098 kb) [file 11671_2018_2600_MOESM1_ESM.docx]

**Additional file 1**

**Electromagnetic energy redistribution in coupled chiral particle chain-film system**

Yuxia Tang^1,3^, Yingzhou Huang^1^, Linhong Qv^2^ and Yurui Fang^2,1,^*

^1^Soft Matter and Interdisciplinary Research Center, College of Physics, Chongqing University, Chongqing, 400044, China.

^2^Key Laboratory of Materials Modification by Laser, Electron, and Ion Beams (Ministry of Education), School of Physics, Dalian University of Technology, Dalian 116024, P. R. China.

^3^School of Computer Science and Information Engineering, Chongqing Technology and Business University, Chongqing, 400067, China

E-mail: Yuxia Tang: [tangyuxia@ctbu.edu.cn](mailto:tangyuxia@ctbu.edu.cn); Yingzhou Huang: [yzhuang@cqu.edu.cn](mailto:yzhuang@cqu.edu.cn); Linhong Qv: [qlh@mail.dlut.edu.cn](mailto:qlh@mail.dlut.edu.cn); Yurui Fang: [yrfang@dlut.edu.cn](mailto:yrfang@dlut.edu.cn)

* correspondence: [yrfang@dlut.edu.cn](mailto:yrfang@dlut.edu.cn)

1. **Optical properties of chiral particle chain-film system with different gaps between nanoparticles**





Figure S1. Optical properties of chiral particle chain-film system with different gaps between nanoparticles (labeled as g). The gaps between NPs-film are still set as 1 nm. The solid curve lines and dotted curve lines represent the spectra for left-hand circular polarization (LCP) and right-hand circular polarization (RCP) exciting light, respectively. (a)The absorption spectra for the different systems with g = 1 nm, g = 2 nm and g = 4 nm. (b) The circular dichroism (CD) spectra for the different systems with g = 1 nm, g = 2 nm and g = 4 nm. The vertical dashed lines from left to right correspond to the resonant peaks at 590 nm, 635 nm, 710 nm and 785 nm, respectively.

1. **Chiral near-field enhancement distributions for different resonant peaks**

To observe the chiral near-field enhancement in the gap between the adjacent two particles, the cross sections which are perpendicular to x-y plane along the two particle center-lines are drawn.


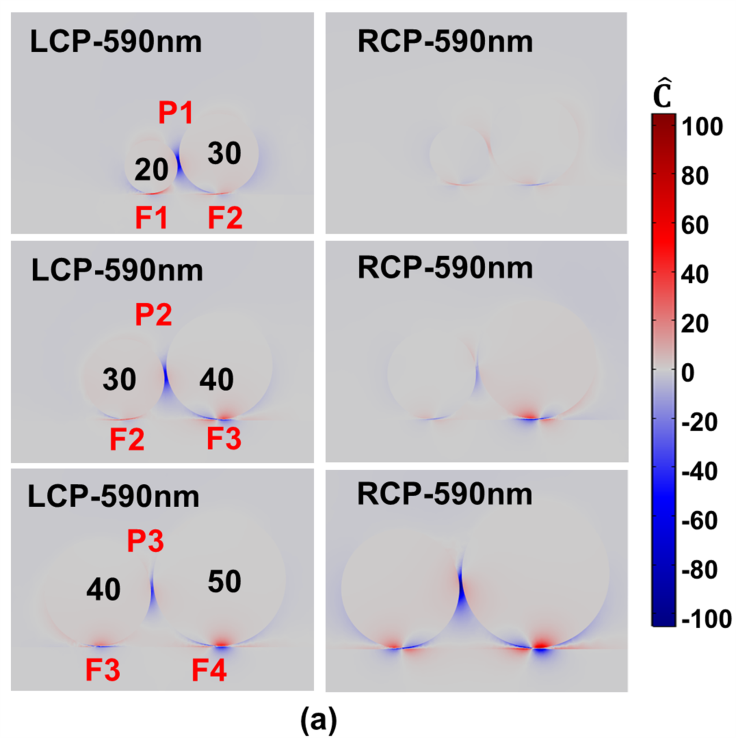


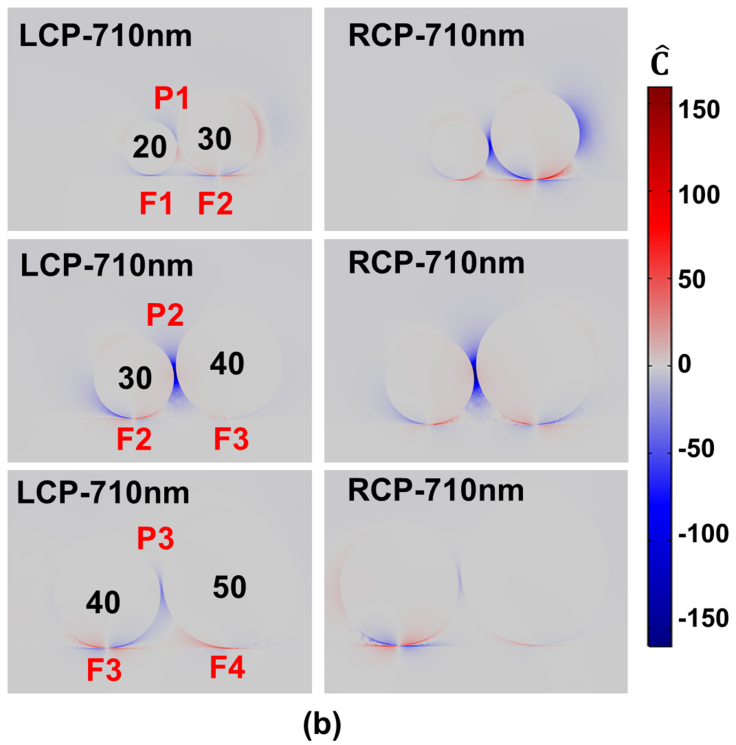


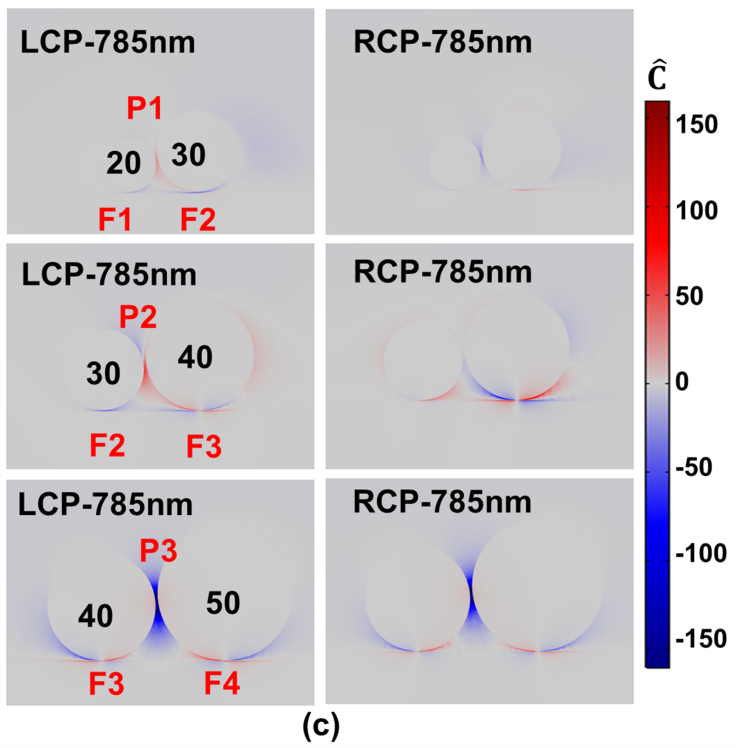


Figure S2. Chiral near-field enhancement distributions at different resonant peaks for LCP and RCP. The resonant wavelengths are (a) 590 nm, (b) 710 nm and (c) 785 nm. The diameters of four silver nanospheres in chiral particle chain-film system are marked as 20 nm, 30 nm, 40 nm and 50 nm, respectively. The gaps between different diameter particles and film are labeled as F1, F2, F3 and F4, respectively; and the gaps between particles are labeled as P1, P2 and P3, respectively.

**3. The optical properties of chiral particle-film system with different R**

To further investigate the effect of the chirality of this structure on optical chirality, we also changed the circle radius (R) in x-y plan (as shown by blue dotted circle in Fig. 1(b)) and simulated the optical properties for different R.





Figure S3. Optical properties of chiral particle chain-film system with different R. R is the radius of the circle shown in Fig.1(b). The values of R in the curves are R=75nm, R=110nm and R=150nm. The gaps between NPs-film and between particles are set as 1 nm and 2 nm, respectively. The radius of four silver NPs in chiral particle chain-film system are 20 nm, 30 nm, 40 nm, and 50 nm, respectively (as shown in Fig. 1). The solid curve lines and dotted curve lines represent the spectra for LCP and RCP light, respectively. (a)The absorption spectra for three systems with different R. (b) The circular dichroism (CD) spectra for these systems. The vertical dashed lines from left to right correspond to the peaks at 590 nm, 635 nm, 710 nm and 785 nm, respectively.

**4. The volume-averaged chiral enhancement spectra between NPs-film in chiral particle-film system with different R**

**

**

Figure S4. Volume-averaged optical chirality enhancement factors between particle-film in the chiral particle chain-film systems with different R. The values of R in the picture are R = 75 nm (blue solid line), R = 110 nm (red solid line) and R = 150 nm (black solid ling). The gaps between NPs-film and between particles are set as 1 nm and 2 nm, respectively. The solid curve lines and dotted curve lines represent the spectra for LCP and RCP light, respectively. The vertical dashed lines from left to right correspond to the peaks at 590 nm, 635 nm, 710 nm and 785 nm, respectively.

**5. The volume-averaged chiral enhancement spectra between particles in chiral particle-film systems with different R**

**

**

Figure S5. Volume-averaged optical chirality enhancement factors between particles in the chiral particle chain-film systems with different R. The values of R in the picture are R = 75 nm (blue solid line), R = 110 nm (red solid line) and R = 150 nm (black solid ling). The gap between NPs-film and between particles are set as 1 nm and 2 nm, respectively. The solid curve lines and dotted curve lines represent the spectra for LCP and RCP light, respectively. The vertical dashed lines from left to right correspond to the peaks at 590 nm, 635 nm, 710 nm and 785 nm, respectively.

**6. Optical properties of chiral particle chain-film systems with different particle numbers**

**

**

**

**

**

**

Figure S6. Optical properties of chiral particle chain-film systems with different particle numbers. The gap between NPs-film and between particles are set as 1 nm and 2 nm, respectively. The solid curve lines and dotted curve lines represent the spectra for LCP and RCP light, respectively. (a) The absorption spectra for two different systems. One of them is the system with four particles discussed above (labeled as 50-40-30-20). The other one is the similar configuration without the 20 nm particle. (labeled as 50-40-30) . (b) The circular dichroism (CD) spectra for 50-40-30 systems. (c) Volume-averaged chiral enhancement between NPs-film for 50-40-30 system. F2, F3 and F4 represent the gaps between the particles of 30 nm, 40 nm and 50 nm and film, respectively. They are consistent with the marks in fig. 2(c). (d) Volume-averaged chiral enhancement between particles for 50-40-30 system. P2 and P3 represent the gaps between 30-40, 40-50 particles, respectively. They are consistent with the marks in the inset of fig. 4. The vertical dashed lines from left to right correspond to the peaks at 590 nm, 635 nm, 710 nm and 785 nm, respectively.
